# Supplementary material for: WhoseEgg: classification software for invasive carp eggs
Source: PeerJ. 2023 Feb 27;11:e14787. doi: 10.7717/peerj.14787 (PMC9979831; doi:10.7717/peerj.14787)
Supplement: Supplemental Information 3 — A PDF generated from the ‘preparing-data-for-app.Rmd’ file with the code used prepare the egg data for the WhoseEgg app along with descriptions and figures. [file peerj-11-14787-s003.pdf]

Preparing Egg Data for WhoseEgg Shiny App

Katherine Goode

Last Updated: July 08, 2021

This notebook contains the code for preparing the egg data for the WhoseEgg app.

Load R packages:

```
library(dplyr)
library(forcats)
library(ggplot2)
library(purrr)
library(randomForest)
library(stringr)
library(tidyr)
library(tools)
```

Identify the Problematic Eggs

We discovered that the manner in which the larval lengths were measured given the egg stage appear to differ between 2014-2015 and 2016. This sections looks more into this and saves a file with all of the possibly problematic eggs.

Load the raw 2014-2016 data from the validation paper:

```
eggdata_csv_pt1 = "https://raw.githubusercontent.com/goodekat/carp-egg-rf-validation/"
eggdata_csv_pt2 = "master/data/eggdata141516_raw.csv"
eggdata_raw <- read.csv(paste0(eggdata_csv_pt1, eggdata_csv_pt2))
```

Clean the data to match what was done to the data for the validation paper (expect for removing extra variables):

```
eggdata_cleaned_all_var <-
  eggdata_raw %>%
  rename_all(
    .funs = function(.) {
      str_replace_all(., "[.]", " ") %>%
        tolower() %>%
        toTitleCase() %>%
        str_replace_all(" ", "_") %>%
        str_replace("Sd", "SD") %>%
        str_replace("Cv", "CV") %>%
        str_replace("Acgc", "ACGC") %>%
        str_replace("Temp", "Temperature")
```

```
    }
  ) %>%
  # Rename variables using yolk to embryo
  rename(
      "Embryo_to_Membrane_Ratio" = "Yolk_to_Membrane_Ratio",
      "Embryo_Ave" = "Yolk_Ave",
      "Embryo_SD" = "Yolk_SD",
      "Embryo_CV" = "Yolk_CV",
  ) %>%
  # Only keep observations with identified genetics
  filter(Questionable_Genetics == "NO") %>%
  # Remove any observations with missing values
  na.omit() %>%
  # Fix some mistakes in site and river levels
  mutate(Site = as.character(Site), River = as.character(River)) %>%
  mutate(Site = ifelse(Site == "DMW", "DNW", Site),
         Site = ifelse(Site == "kqa", "KQA", Site),
         River = ifelse(River == "UNR", "UMR", River),
         River = ifelse(River == "UPR", "UMR", River),
         River = ifelse(River == "DMS", "DSM", River)) %>%
  mutate(Site = factor(Site), River = factor(River))
```

Print the dimensions of the cleaned data:

```
dim(eggdata_cleaned_all_var)
```

```
## [1] 1979   46
```

Create a table showing the proportion of observations with a larval length of 0 for each egg stage and year – note that in 2014 and 2015, all eggs that were not in egg stage 7 or 8 have a larval length of 0, and all eggs in egg stage of 7 or 8 have a larval of 0 – this is not the case in 2016:

```
eggdata_cleaned_all_var %>%
  group_by(Egg_Stage, Year) %>%
  summarise(
    n_zeros = sum(Larval_Length == 0),
    freq = n(),
    .groups = "drop"
  ) %>%
  mutate(prop_of_zeros = round(n_zeros / freq, 4)) %>%
  select(-n_zeros, -freq) %>%
  pivot_wider(id_cols = Egg_Stage, names_from = Year, values_from = prop_of_zeros) %>%
  knitr::kable()
```

Egg_Stage	2014	2015	2016
1	1	1	1.0000
2	1	1	0.9000
3	1	1	0.9744
4	1	1	1.0000
5	1	1	0.9836
6	1	1	0.7975

Egg_Stage	2014	2015	2016
7	0	0	0.1346
8	0	0	0.0345
BROKEN	1	NA	NA
D	1	1	0.9796

Identify the eggs from 2016 with non-zero larval lengths with an egg stage other than 7 or 8:

```
eggs_wrong_non_zero_lls <-
  eggdata_cleaned_all_var %>%
  filter(Year == 2016, Egg_Stage %in% c(1:6, "D", "BROKEN"), Larval_Length > 0)
```

Identify the eggs from 2016 with larval lengths of zero with an egg stage of 7 or 8:

```
eggs_wrong_zero_lls  <-
  eggdata_cleaned_all_var %>%
  filter(Year == 2016, Egg_Stage %in% c(7,8), Larval_Length == 0)
```

Join the possibly problematic eggs:

```
eggs16_problems <-
  bind_rows(eggs_wrong_non_zero_lls, eggs_wrong_zero_lls)
```

Print the dimensions of the possibly problematic eggs:

```
dim(eggs16_problems)
```

```
## [1] 29 46
```

Save the possibly problematic eggs:

```
write.csv(eggs16_problems, "../data/problematic-eggs.csv", row.names = FALSE)
```

Comments from Mike About Problematic Eggs

- Egg stages from the problematic eggs all look okay
- For the eggs in stages other than 7 and 8, we could change the larval lengths to 0 to match Carlos's approach
- For the eggs in stages 7-8, he can't get the larval lengths
- Should we take all of these eggs out?
- Should we remove only the eggs in stages 7 and 8 with non-zero larval lengths
- Try fitting models and see how it affects the results from the various options
- Mike's email with updated eggs: "A lot of the rest I looked at I changed to stage 7, so the length was valid. I put a few comments in the column at the end." Updated data is in the file "2016 eggs with incorrect data_MW.csv"

Comparing Models

Here I create four different datasets and fit a model for each to compare how handling the problematic eggs affects the results. The four datasets/models I consider are the full dataset from the validation paper with problematic eggs:

- unchanged
- removed
- corrected by Mike
- corrected by Mike but eggs in stage 7 and 8 with larval lengths of 0 removed

Preparation

Make a list of the response variables:

```
vars_resp = c(
  "Family_ACGC",
  "Genus_ACGC",
  "Common_Name_ACGC"
)
```

Make a vector of the predictor variables:

```
vars_pred = c(
  "Month",
  "Julian_Day",
  "Temperature",
  "Conductivity",
  "Larval_Length",
  "Membrane_Ave",
  "Membrane_SD",
  "Membrane_CV",
  "Embryo_to_Membrane_Ratio",
  "Embryo_Ave",
  "Embryo_SD",
  "Embryo_CV",
  "Egg_Stage",
  "Compact_Diffuse",
  "Pigment",
  "Sticky_Debris",
  "Deflated"
)
```

Function for preparing the data to match the form from the validation paper:

```
clean_eggdata <- function(data) {
  data %>%
    # Convert necessary variables to factors
    mutate_at(
      .vars = c(
        "Egg_Stage",
        "Compact_Diffuse",
```

```
        "Pigment",
        "Sticky_Debris",
        "Deflated",
        all_of(vars_resp)
      ),
      .funs = factor
    ) %>%
    # Make sure the Invasive Carp level is still the first factor level (like ACGC was)
    # Otherwise, the random forest results will change slightly
    mutate(
      Family_ACGC = fct_relevel(Family_ACGC, "Invasive Carp"),
      Genus_ACGC = fct_relevel(Genus_ACGC, "Invasive Carp"),
      Common_Name_ACGC = fct_relevel(Common_Name_ACGC, "Invasive Carp")
    )
}
```

Reconstruct Data from Validation Paper

Load the data from the validation paper and prepare as necessary for analysis:

```
eggdata_val_paper <-
  # Access the data from the validation paper that will be used for WhoseEgg
  # The difference from the data accessed earlier is the lack of some extra
  # variables that are not needed for the validation paper or WhoseEgg
  read.csv(
    paste0(
      "https://raw.githubusercontent.com/goodekat/",
      "carp-egg-rf-validation/master/data/eggdata141516.csv"
    )
  ) %>%
  # Change the level of ACGC to Invasive Carp for easier terminology in the app
  mutate(
    Family_ACGC = fct_recode(Family_ACGC, "Invasive Carp" = "ACGC"),
    Genus_ACGC = fct_recode(Genus_ACGC, "Invasive Carp" = "ACGC"),
    Common_Name_ACGC = fct_recode(Common_Name_ACGC, "Invasive Carp" = "ACGC")
  ) %>%
  # Finish cleaning steps
  clean_eggdata() %>%
  # Remove unnecessary variable
  select(-Dataset)
```

Create Data with All Problem Observation Removed

Identify the eggs from 2016 with non-zero larval lengths with an egg stage other than 7 or 8 and the eggs from 2016 with larval lengths of zero with an egg stage of 7 or 8:

```
eggdata_flagged <-
  eggdata_val_paper %>%
  mutate(Flagged = ifelse(
    Year == 2016 & Egg_Stage %in% c(1:6, "D", "BROKEN") & Larval_Length > 0,
    TRUE,
```

```
    ifelse(Year == 2016 & Egg_Stage %in% c(7, 8) & Larval_Length == 0,
           TRUE,
           FALSE)
  )) %>%
  select(Flagged, everything())
```

Determine the number of problematic (flagged) eggs:

```
eggdata_flagged %>% count(Flagged)
```

```
##   Flagged    n
## 1   FALSE 1949
## 2    TRUE   29
```

Remove the problem observations:

```
eggdata_problems_removed <- eggdata_flagged %>% filter(Flagged == FALSE)
```

Check that the correct number of observations have been removed:

```
n_removed_eggs = (dim(eggdata_val_paper)[1] - dim(eggdata_problems_removed)[1])
n_problem_eggs = table(eggdata_flagged$Flagged)[[2]]
n_removed_eggs == n_problem_eggs
```

```
## [1] TRUE
```

Create Data with Egg Stages Corrected by Mike

Load the data with the problematic eggs with egg stage and larval length corrected by Mike where possible and clean as needed:

```
eggs_corrected_MW <-
  read.csv("../data/2016 eggs with incorrect data_MW.csv") %>%
  # Convert variables to numeric as needed
  mutate(Conductivity = as.numeric(Conductivity)) %>%
  # Change the level of ACGC to Invasive Carp for easier terminology in the app
  mutate(
    Family_ACGC = fct_recode(Family_ACGC, "Invasive Carp" = "ACGC"),
    Genus_ACGC = fct_recode(Genus_ACGC, "Invasive Carp" = "ACGC"),
    Common_Name_ACGC = fct_recode(Common_Name_ACGC, "Invasive Carp" = "ACGC")
  ) %>%
  # Finish cleaning steps
  clean_eggdata()
```

Identify the variable names to keep for the analysis based on those in the validation paper data:

```
var_names = names(eggdata_val_paper)
```

Select the problematic eggs from the validation data and the correct data and check that all observations are the same (except for egg stage and larval length):

```r
# Get the validation paper data problematic eggs
problem_eggs_old <-
  eggdata_flagged %>%
  filter(Flagged == TRUE) %>%
  select(-Flagged,-Egg_Stage,-Larval_Length) %>%
  mutate(
    Family_ACGC = factor(Family_ACGC),
    Genus_ACGC = factor(Genus_ACGC),
    Common_Name_ACGC = factor(Common_Name_ACGC)
  )

# Get the corrected versions of the problematic eggs
problem_eggs_corrected <-
  eggs_corrected_MW %>%
  select(all_of(var_names)) %>%
  select(-Egg_Stage, -Larval_Length)

# Check that the other variables are the same
all_equal(problem_eggs_old, problem_eggs_corrected)
```

```
## [1] TRUE
```

Join the good eggs from the validation paper data with the corrected versions of the problem eggs and clean data as necessary:

```r
eggdata_corrected <-
  eggdata_problems_removed %>%
  bind_rows(eggs_corrected_MW %>% select(all_of(var_names))) %>%
  clean_eggdata() %>%
  select(-Flagged)
```

Perform some checks to make sure the data dimensions are correct:

```r
# Check that there are no eggs with egg stage not in 7 or 8 with a larval length above 0
dim(eggdata_corrected %>% filter(!(Egg_Stage %in% c(7,8)), Larval_Length > 0))[1] == 0
```

```
## [1] TRUE
```

```r
# Check that the number of observations is correct
dim(eggdata_corrected)[1] == dim(eggdata_val_paper)[1]
```

```
## [1] TRUE
```

Create Data with Egg Stages Corrected by Mike and Unknown Observations Removed

There are still some eggs in the data in stages 7 and 8 with a larval length of 0. Mike was not able to fix the larval length for these eggs (due to eggs already having been destroyed and the pictures not providing good angles for measurement):

```
dim(eggdata_corrected %>% filter(Egg_Stage %in% c(7,8), Larval_Length == 0))[1]
```

```
## [1] 6
```

Remove the eggs in stages 7 and 8 with larval lengths of 0 and clean data as necessary:

```
eggdata_corrected_removed <-
  eggdata_corrected %>%
  filter(!(Egg_Stage %in% c(7,8)) | Larval_Length != 0) %>%
  clean_eggdata()
```

Fit All Random Forests

Function for fitting a random forest model given a response variable, predictor variables, and a dataset (uses the same seed to fit the random forests as Camacho et al. (2019) and Goode et al. (2021)):

```
fit_rf <- function(resp, preds, data) {

  # Fit the random forest
  set.seed(808)
  rf <- randomForest(
    data %>% pull(resp) ~ .,
    data = data %>% select(all_of(preds)),
    importance = T,
    ntree = 1000
  )

  # Put model in a named list
  rf_list = list(rf)
  names(rf_list) = resp

  # Return the named list
  return(rf_list)

}
```

Fit the random forest models:

```
rfs_val_data <-
  map(
    .x = vars_resp,
    .f = fit_rf,
    preds = vars_pred,
    data = eggdata_val_paper
  ) %>%
  flatten()

rfs_prob_remv <-
  map(
    .x = vars_resp,
    .f = fit_rf,
```

```
    preds = vars_pred,
    data = eggdata_problems_removed
  ) %>%
  flatten()

rfs_prob_corr <-
  map(
    .x = vars_resp,
    .f = fit_rf,
    preds = vars_pred,
    data = eggdata_corrected
  ) %>%
  flatten()

rfs_prob_corr_remv <-
  map(
    .x = vars_resp,
    .f = fit_rf,
    preds = vars_pred,
    data = eggdata_corrected_removed
  ) %>%
  flatten()
```

Check to make sure the random forests trained on the unchanged data from the validation paper agree (note that these random forests are available on GitHub)

```
rfs141516 <- readRDS("../../../validation/results/rfs141516.rds")
c(
  identical(rfs141516$Family_ACGC$forest, rfs_val_data$Family_ACGC$forest),
  identical(rfs141516$Genus_ACGC$forest, rfs_val_data$Genus_ACGC$forest),
  identical(rfs141516$Common_Name_ACGC$forest, rfs_val_data$Common_Name_ACGC$forest)
)
```

```
## [1] TRUE TRUE TRUE
```

Compute Random Forest Metrics

Functions for computing metrics from the validation paper:

```
# Function for computing predictive accuracy
compute_pa <- function(category, obs, pred) {
  data.frame(obs, pred) %>%
    filter(obs == category) %>%
    summarise(pa = sum(obs == pred) / n()) %>%
    as.numeric()
}
# Function for computing false positive accuracy
compute_fpr <- function(category, obs, pred) {
  data.frame(obs, pred) %>%
    filter(obs != category) %>%
    summarise(sum(pred == category) / n()) %>%
    as.numeric()
```

```
}
# Function for computing non-target taxa accuracy
compute_prec <- function(category, obs, pred) {
 data.frame(obs, pred) %>%
    filter(pred == category) %>%
    summarise(sum(obs == pred) / n()) %>%
    as.numeric()
}
```

Function for applying the metrics to the models in this notebook using out-of-bag (OOB) predictions:

```
# Function for computing validation metrics
compute_metrics <- function(scenario, model, model_name, data) {

  # Extract the response variable from the validation data
  obs = data %>% pull(model_name)

  # Apply the random forest model to the validation data
  # to obtain predictions
  pred = predict(object = model)

  # Compute the metrics for validation for each class and put them
  # in a data frame
  class = "Invasive Carp"
  data.frame(
    scenario = scenario,
    model = model_name,
    class = class,
    pa = compute_pa(class, obs = obs, pred = pred),
    fpr = compute_fpr(class, obs = obs, pred = pred),
    prec = compute_prec(class, obs = obs, pred = pred)
  ) %>%
    mutate_at(.vars = c("model", "class"), .funs = as.character)

}
```

Compute the OOB metrics from all models on their respective training data:

```
metrics_oob <-
  bind_rows(
    map2_df(
      .x = rfs_val_data,
      .y = names(rfs_val_data),
      .f = compute_metrics,
      data = eggdata_val_paper,
      scenario = "validation_paper"
    ),

    map2_df(
      .x = rfs_prob_remv,
      .y = names(rfs_prob_remv),
      .f = compute_metrics,
      data = eggdata_problems_removed,
```

```r
      scenario = "problems_removed"
    ),

    map2_df(
      .x = rfs_prob_corr,
      .y = names(rfs_prob_corr),
      .f = compute_metrics,
      data = eggdata_corrected,
      scenario = "problems_corrected"
    ),

    map2_df(
      .x = rfs_prob_corr_remv,
      .y = names(rfs_prob_corr_remv),
      .f = compute_metrics,
      data = eggdata_corrected_removed,
      scenario = "corrected_and_removed"
    )
  )

metrics_oob
```

```
##                 scenario            model         class        pa        fpr
## 1       validation_paper      Family_ACGC Invasive Carp 0.9731458 0.03511706
## 2       validation_paper       Genus_ACGC Invasive Carp 0.9757033 0.04096990
## 3       validation_paper Common_Name_ACGC Invasive Carp 0.9808184 0.04096990
## 4       problems_removed      Family_ACGC Invasive Carp 0.9717587 0.03589744
## 5       problems_removed       Genus_ACGC Invasive Carp 0.9781772 0.04017094
## 6       problems_removed Common_Name_ACGC Invasive Carp 0.9820282 0.04102564
## 7     problems_corrected      Family_ACGC Invasive Carp 0.9731458 0.03762542
## 8     problems_corrected       Genus_ACGC Invasive Carp 0.9731458 0.03678930
## 9     problems_corrected Common_Name_ACGC Invasive Carp 0.9808184 0.04013378
## 10 corrected_and_removed      Family_ACGC Invasive Carp 0.9757033 0.03529412
## 11 corrected_and_removed       Genus_ACGC Invasive Carp 0.9744246 0.03949580
## 12 corrected_and_removed Common_Name_ACGC Invasive Carp 0.9820972 0.04033613
##         prec
## 1  0.9476961
## 2  0.9396552
## 3  0.9399510
## 4  0.9474343
## 5  0.9419036
## 6  0.9409594
## 7  0.9441687
## 8  0.9453416
## 9  0.9411043
## 10 0.9478261
## 11 0.9419036
## 12 0.9411765
```

Plot the metrics:

```r
metrics_oob %>%
  pivot_longer(cols = c(pa, fpr, prec), names_to = "metric") %>%
  mutate(
    metric = fct_recode(
      metric,
      "False positive rate" = "fpr",
      "Predictive accuracy" = "pa",
      "Precision" = "prec"
    ),
    scenario = fct_relevel(
      scenario,
      "validation_paper",
      "problems_removed",
      "problems_corrected",
      "corrected_and_removed"
    )
  ) %>%
  ggplot(aes(
    x = model,
    y = value,
    group = scenario,
    linetype = scenario,
    color = scenario
  )) +
  geom_point() +
  geom_line(size = 1, alpha = 0.75) +
  facet_grid(metric ~ ., scales = "free_y") +
  scale_color_manual(values = wesanderson::wes_palettes$Darjeeling1) +
  theme_bw() +
  labs(
    x = "Model Response Variable",
    y = "Metric Value",
    color = "",
    linetype = "",
    title = "Metrics Computed for Prediction Class of Invasive Carp"
  )
```

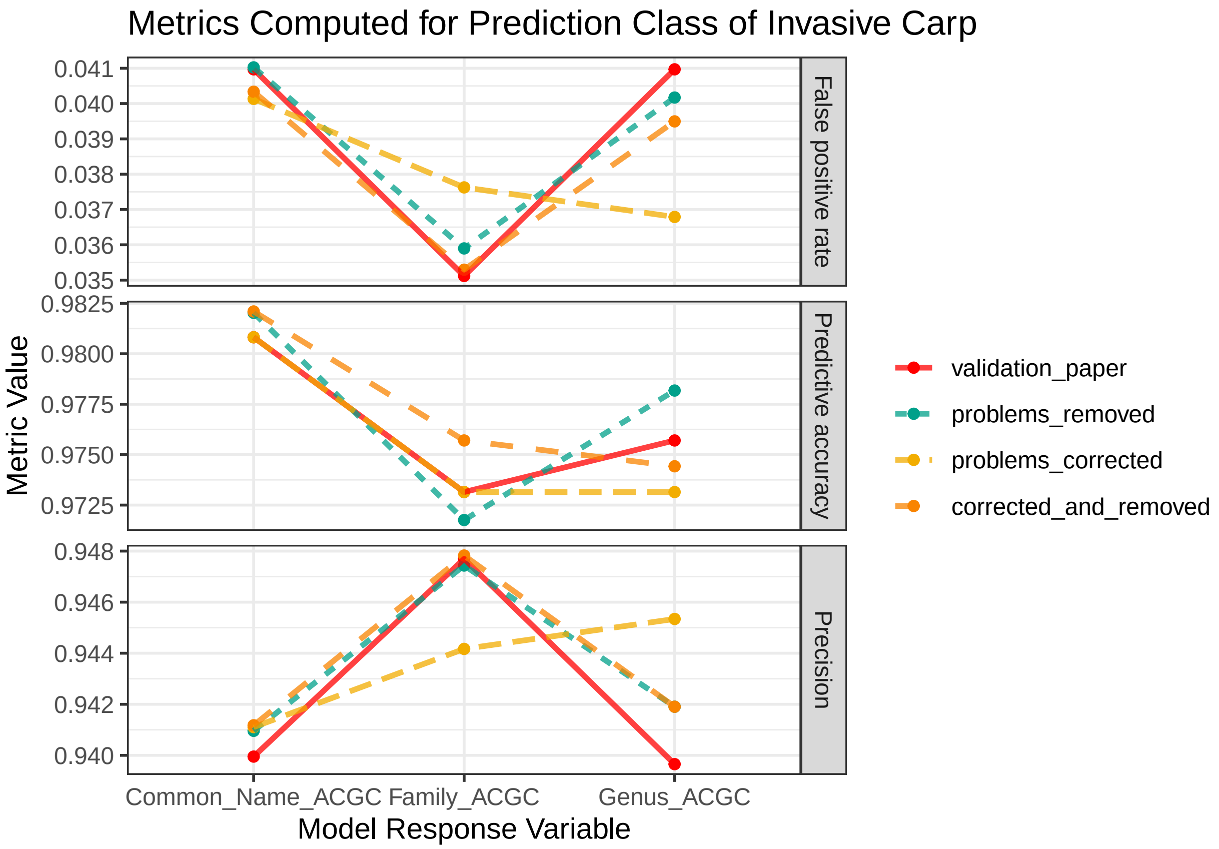

Save the Egg Data for WhoseEgg

Since the metric results were not very different across the models trained on the four datasets, we decided to use the dataset that is as accurate as possible: "corrected_and_removed". That is, the data includes the corrections made by Mike, and the observations that are in egg stage 7 or 8 with a larval length of 0 have been removed.

Perform some additional cleaning on the data:

```
eggdata_for_app <-
  eggdata_corrected_removed %>%
  rename(Family_IC = Family_ACGC, Genus_IC = Genus_ACGC, Common_Name_IC = Common_Name_ACGC)
```

Save the data for WhoseEgg:

```
write.csv(
  x = eggdata_for_app,
  file = "../data/eggdata_for_app.csv",
  row.names = FALSE
)
```

Session Info

```r
sessionInfo()
```

```
## R version 4.0.4 (2021-02-15)
## Platform: x86_64-apple-darwin17.0 (64-bit)
## Running under: macOS Big Sur 10.16
##
## Matrix products: default
## BLAS:   /Library/Frameworks/R.framework/Versions/4.0/Resources/lib/libRblas.dylib
## LAPACK: /Library/Frameworks/R.framework/Versions/4.0/Resources/lib/libRlapack.dylib
##
## locale:
## [1] en_US.UTF-8/en_US.UTF-8/en_US.UTF-8/C/en_US.UTF-8/en_US.UTF-8
##
## attached base packages:
## [1] tools     stats     graphics  grDevices utils     datasets  methods
## [8] base
##
## other attached packages:
## [1] tidyr_1.1.3         stringr_1.4.0       randomForest_4.6-14
## [4] purrr_0.3.4         ggplot2_3.3.3       forcats_0.5.1
## [7] dplyr_1.0.6
##
## loaded via a namespace (and not attached):
##  [1] pillar_1.6.1      compiler_4.0.4    highr_0.9         digest_0.6.27
##  [5] evaluate_0.14     lifecycle_1.0.0   tibble_3.1.2      gtable_0.3.0
##  [9] pkgconfig_2.0.3   rlang_0.4.11      DBI_1.1.1         yaml_2.2.1
## [13] xfun_0.23         withr_2.4.2       knitr_1.33        generics_0.1.0
## [17] vctrs_0.3.8       grid_4.0.4        tidyselect_1.1.1  glue_1.4.2
## [21] R6_2.5.0          fansi_0.5.0       wesanderson_0.3.6 rmarkdown_2.9
## [25] farver_2.1.0      magrittr_2.0.1    scales_1.1.1      ellipsis_0.3.2
## [29] htmltools_0.5.1.1 assertthat_0.2.1  colorspace_2.0-1  labeling_0.4.2
## [33] utf8_1.2.1        stringi_1.6.2     munsell_0.5.0     crayon_1.4.1
```
